# Vertical Greening Systems: A Critical Comparison of Do-It-Yourself Designs

**DOI:** 10.3390/plants11233230

**Published:** 2022-11-25

**Authors:** Laura Dominici, Elena Comino, Fraser Torpy, Peter Irga

**Affiliations:** 1 Department of Environment, Land and Infrastructure Engineering, Politecnico di Torino, 10129 Turin, Italy; 2 Faculty of Science, School of Life Sciences, University of Technology Sydney, Sydney 2007, Australia; 3 Faculty of Engineering and Information Technology, School of Civil and Environmental Engineering, University of Technology Sydney, Sydney 2007, Australia

**Keywords:** urban greening, Do-It-Yourself, green walls, community awareness, co-design processes, vertical greening systems

## Abstract

Due to the increasing shortage of space in urban areas, vertical greening systems (VGSs) are becoming increasingly popular as a means to provide increased urban greening using building façades. VGSs are usually installed and managed by experts due to technical complexity, however the role of local communities is becoming increasingly important through Do-It-Yourself (DIY) practices. This study aims to explore low-cost VGSs and provide design suggestions and maintenance indications to encourage the expanded use of in situ small-scale VGSs. Firstly, an exploratory review of VGS designs proposed in the scientific literature, and by commercial and community-based solutions was conducted taking DIY potential into account to define eight basic design models categorized through six structural criteria. Then, seven community garden groups were interviewed to inform a critical comparison of the eight design models. Data collected was synthesized to develop a star rating system, thus providing a quick comparative tool. The star rating system shows the performance of five relevant DIY design parameters for each VGS model. The current research may assist in the accessibility of green technologies and facilitate community-scale implementation of DIY vertical greening.

## 1. Introduction

Increasing population densities in urban areas will require the reconsideration of the structure of cities, along with building requirements to pursue committed and strategic actions to increase the livability of the built environment. Green urban areas are considered essential places that support people’s physical and mental health and wellbeing [1], however urbanization and land-use changes put public green areas under increasing pressure [2]. The UN Sustainable Development Goal No. 11: Make cities inclusive, safe, resilient and sustainable [3] introduces the concept of Green Infrastructures (GI) and Nature-based Solutions (NBSs) as strategies to design more sustainable cities. NBSs are applied to address environmental challenges within urban contexts, whilst additionally providing social and economic benefits [4,5]. GIs are described as a network of multifunctional green spaces covered by vegetation, such as parks, green corridors, private and public gardens, green roofs and green walls [6]. GIs harness NBSs in urban areas to deliver ecologically sound outcomes [7], which have been recognized by both scientists and politicians to improve city habitability [8].

Urban horticulture is a form of NBS that can contribute to supporting mental health and wellbeing [9], and form part of a systemic approach to face emerging societal challenges [10]. Urban agriculture phenomenon offers the opportunity to transform urban space and promote place-making for social purposes in both high and low-income countries [11]. The benefits notwithstanding, the move towards urban gardening and the re-greening of cities is constrained due to the lack of space in cities with high density populations [12], as well as soil contaminations and public safety concerning home gardening and urban farming [13]. This has led to an increase in interest in alternative GI technologies; notably green roofs and vertical greening systems (VGSs), which allow for the space-efficient integration of vegetated surfaces in urban areas [14]. A growing body of evidence suggests VGS technology can improve air quality [15], mitigate the urban heat island effect [16], improve building performance by acting as thermal insulation [17], support biodiversity in cities [18] and manage stormwater [19]. In some cases, VGSs are adopted as NBSs used to revitalize and regenerate urban vacant lands [20] and to promote a biophilic urbanisms [21].

However, as VGS technology is relatively new, and proprietary systems require substantial technical knowledge to install and manage, the feasibility of implementing VGS at a community scale is often challenging. In many cases, the most effective vertical greening initiatives are managed by local governments which collaborate with citizens and private sector to foster the implementation of community-scale and localized interventions [22]. Rupp et al. [23] demonstrated that highly intensive civic engagement and active participation in planning and implementing urban greening results in more effective and accepted interventions.

In this framework, Do-It-Yourself (DIY) urban greening initiatives are frequently adopted by citizens to add greenery to urban environments and improve the surrounding built environment [24]. DIY urban greening refers to the practice in which non-expert community’s members create or repurpose urban spaces using non-professional materials and processes [25]. DIY urban greening as an informal civic initiative could increase citizens’ participation in the process of urban sustainable transition, and promote community empowerment and social inclusiveness [26]. DIY activities could also promote VGSs as educative tools [27] for the community-based social and ecological transformation of urban spaces. However, the public understanding of achievable DIY VGS designs and the technical considerations involved is lacking, as are recommendations on designs that take into account the various types of structure or irrigation systems, which will change depending on the motivation for each VGS application. All these aspects influence the sustainability and cost-effectiveness of VGSs, which also differ case by case. In many cases, technical requirements are inaccessible to the public or not known at all, because they are usually targeted at experts who design, construct, install and maintain vertical greening professionally. These factors are highly reliant on various economic, technical, and environmental influences, as well as commitment and engagement, which will also impact the effectiveness of the system and user satisfaction. Therefore, the design process of a VGS is crucial to create a sustainable and successful greening solution which meets community needs and limitations [28]. Decision-making for designing the most appropriate VGS could be challenging for inexpert individuals or communities and sharing knowledge can support successful co-design processes that involve local governments, private sector and citizens [29].

In order to make more accessible vertical green wall technologies in the DIY urbanism [30], this study reports on and evaluates low cost product designs to encourage VGS applications in a low-income urban settlement or at a residential scale. Due to the complex and multifaceted nature of VGSs, this study is divided into two stages: (1) the analysis of different technical solutions based on a review of the published literature, on the most common commercial solutions and DIY systems and the experience of local stakeholders; (2) a critical comparison between VGS design models using a simple rating system to assess DIY performance. Although previous studies have focused on technical features to improve the ornamental and functional role of VGSs, the aim of this work was to provide a design guideline for individuals and communities who want to install low-budget or small-scale green walls. The guideline consists of a set of safe design considerations, maintenance indications and planting recommendations provided to users in order to promote positive experiences of VGSs because it’s a relatively new greening technology and it will be developed step by step [31]. The rating system approach aims to provide user-friendly design information based on priorities and needs expressed by non-expert individuals or communities for implementing green walls as urban greening tools. Thus, the investigations’ goal was not to provide quantitative data, but rather to provide qualitative analysis based on community engagement.

### An Introduction to VGSs for the Greening Improvement in Urban Areas 

The concept of green walls or vertical greening systems (VGS) applies to all systems that can sustain vegetation that grow vertically on, up or within a surface, such as façades or walls, without any or with limited ground level space use [32]. These systems partially or completely cover the building wall with supporting structures for vegetation, which may include a plant growth medium. Although the use of VGS is an old design practice for greening cities [33,34]), new technological solutions are becoming more frequently applied to VGSs to address sustainability in urban areas [35]. Overall, the terms ‘vertical greening system’, or ‘vertical garden’ can be seen as over-arching umbrella terms used to describe all forms of vegetated wall surfaces [36,37]. Throughout the green infrastructure literature, different green wall proponents have adopted a variety of definitions, classification systems and terminology. Pérez & Perini [38] generally categorised VGSs into two different groups: green façades and living wall systems (LWS). Green façades are the most traditional VGSs [39], which utilises hanging or climbing plants, such as lianas, vines or scramblers, as vegetation cover. A common characteristic to identify these systems are plants rooted at the base of walls or in planter boxes, which can be attached to the wall at a height or at the base. Green façades were categorised as either direct or indirect designs [40], according to the location of the vegetation, either directly attached to the wall or supported by structures to allow plants to climb and spread. 

Comparatively, LWSs were recently introduced to increase the variety of plants that can be cultivated vertically, with the aim to obtain a more uniform vegetated surface in high buildings [36]. LWSs can be “self-sustained”, also known as “free-standing”, or “wall-attached” systems, and they may be structured as pre-vegetated modular fixtures or continuous pocketed frames, which are attached to the wall. Both structures—modular and continuous—are indirect systems that contain and isolate the plant growth media from the building wall surface. Therefore, LWSs, as self-sufficient systems, differ from green façades by allowing plant growth without the need for rooting into the natural ground surface. Despite their ornamental values, LWSs require the use of expensive materials and frequent management, often affecting their cost effectiveness and applicability in any context [41]. Additionally, they require specific technical knowledge to select the most appropriate plant species and frequent maintenance interventions. 

Modular LWSs include a huge variety of systems that differ in structure, weight, number of components and assembling complexity, being are designed to improve the flexibility and adaptability of VGSs to user’s needs. For that reason, only modular VGS structures have been considered and analysed for DIY applications. 

## 2. Methodology

### 2.1. Categorisation of VGS Models for DIY Application

An exploratory literature search of existing VGS typology was conducted in order to explore VGS models that have DIY potential (from design stage until maintenance) and could be integrated into a community’s decision-making process in urban design and gardening. The search was based on VGS technical considerations identified in the published literature, existing market products, online DIY tutorials and community garden experiences. The academic databases, ScienceDirect and Science Research, as well as the online search engines Google Scholar, Academia and Research Gate, were searched to identify projects adopted for community purposes all over the world. Additionally, the informal social media platform such as Youtube and Pinterest were also examined for related DIY initiatives. 

Existing VGSs are classified based on criteria defined by previous studies, based on construction characteristics [36,37,42] and are described based on the following criteria: (1) structure, material and components; (2) irrigation and drainage systems; (3) type of vegetation; (4) indoor or outdoor application; (5) maintenance requirements; (6) aesthetic value. This allowed for the synthesis of VGS design models which are DIY focused based on these criteria. Principal positive and negative aspects of each design model were also determined as below.

### 2.2. Comparison between VGS Design Models

A comparative star rating system was created to rank designs for various applications, with the goal to assist communities or individuals in the selection of the most appropriate VGS design model among those identified during the categorisation stage. The intention was not only to benchmark the perceived performance of VGS design models, but also to communicate key information to inexpert makers. 

**Figure 1:**
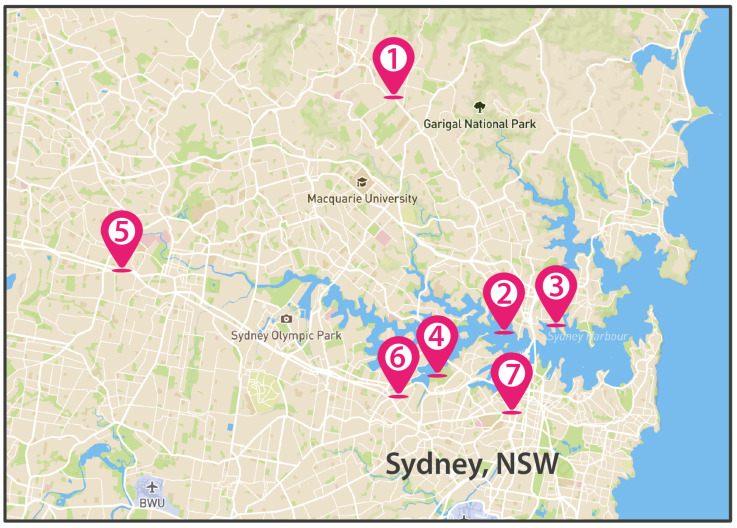
City map of Sydney (NSW, Australia) with location of community garden groups (their names are reported in Table 1), modified from [43].

The methodology was based on the participatory engagement of representative members (ranging from 1–3 people) from seven community garden groups located in Sydney Region (Sydney, New South Wales, Australia) who were interviewed for this research (locations in Figure 1 and Table 1). The community garden groups engaged in this investigation have the largest membership in the Sydney Region. Additionally, the representative members were deemed to be ‘senior’ or high ranking in their respective community groups. In the first instance, interviews were conducted to confirm the DIY appropriateness of the designs, and subsequently to provide qualitative data enabling the comparison ratings assigned to each of eight VGS designs. The participatory engagement was useful to explore the local-scale scenario of urban and sub-urban community gardens and to prioritise the main needs and challenges expressed by stakeholders.

Five main criteria were identified for inclusion into the rating tool, which were identified through semi-informal interviews with community garden stakeholders based on their own experiences: (1) DIY friendliness, (2) cost effectiveness, (3) integration with existing buildings, (4) maintenance, (5) drainage and irrigation. These criteria have been selected with a priority of addressing major community needs and limitations, thus facilitating the selection of the most appropriate design model. At least three stakeholders from each community garden group were asked to give a score from one to five for each criterion for each VGS model based on their own experience. The evaluation process for each criterion has been guided by the qualitative descriptive questions shown in Table 2. The scores were then converted into a comparative star rating system, which allows for a user friendly evaluation to facilitate the selection of the most suitable and achievable VGS design for the needs of an individual or community group. Stakeholders were involved separately during semi-informal interviews in order to outline differences between community gardens.

## 3. Analysis and Results

### 3.1. Categorisation of VGS Models for DIY Application

**Figure 2:**
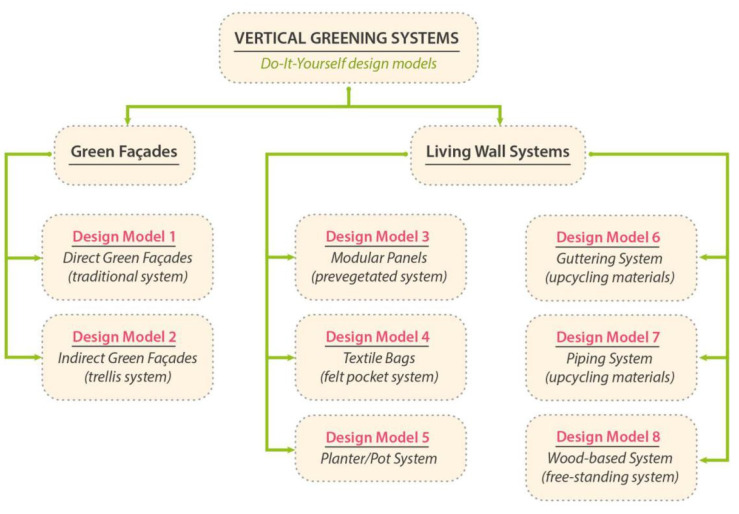
Categorization of VGS design models for DIY applications based on previous study classification.

Using the classification definitions outlined by Manso & Castro-Gomes [36] and Radosavljevic et al. [44], the proposed DIY VGSs were classified into eight design categories, maintaining the distinction between green façades and LWS (Figure 2).

### 3.2. Design Models 1 & 2: Features of Green Façades

**Figure 3:**
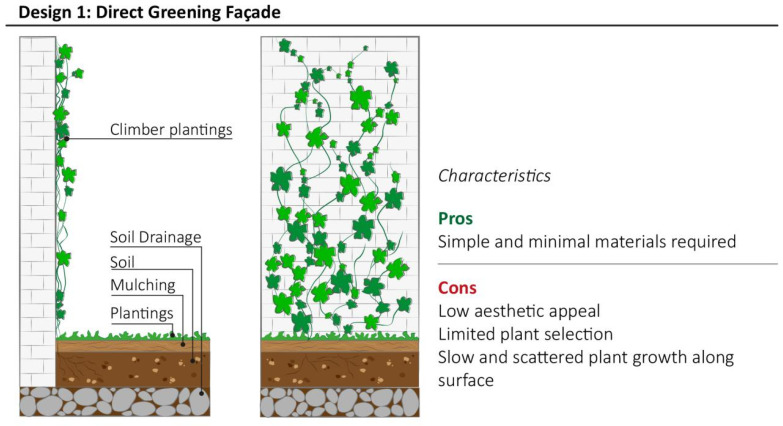
Features of Direct Green Façade with the summary of most relevant characteristics highlighted by community representatives during interviews.

Direct green façades (Design model 1, Figure 3) take inspiration from ancient architecture techniques from the Mediterranean region and Central Europe of covering palace façades with vines and climbing plants that became popular in Berlin (Germany) between 1980 and 1997 [33]. Design model 1 is the simplest design and was the most low-cost model of VGS [45] which can be easily implemented in high density urban areas, especially in outdoor environments, due to the limited number of materials required to build it. 

**Figure 4:**
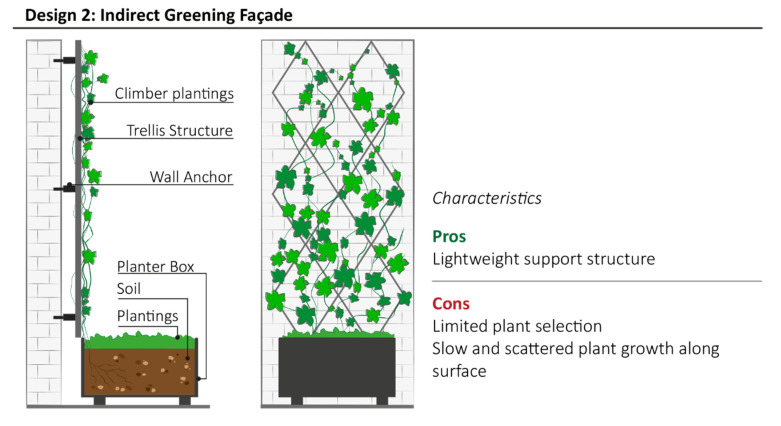
Schematic structure of Indirect Green with the summary of most relevant characteristics highlighted by community representatives during interviews.

Design model 2 uses vertical support structures; such as bamboo, wood, steel, aluminum or HDPE; as a trellis to guide plant growth, and in turn, increase coverage of the building surface and reduce the risk of VGS collapse (Figure 4). This VGS model is often applied on residential fences and commercial building facades as low-budget greening solution. Modular or continuous trellis, mesh, nets, wires or cables, running horizontally or vertically assist and control plant growth, providing an anchor for plants to grasp and attach to. It is also known as a “double-skin façade” because the vertical vegetated structure creates an air gap with the building surface to preserve the integrity.

Vegetation choice is the most limiting factor for the implementation of Design models 1 and 2. Direct green façades require self-clinging climber plant species, such as *Hedera helix*, *Parthenocissus tricuspidata*, *Wisteria* sp. and *Vitis* sp., which utilise adhesive pads or clinging aerial rootlets to attach and spread on a wall surface. Design model 1 is a self-supporting system that requires a medium–long period to cover large areas of building surface, depending on the plant species used. Indeed, evergreen plant species should be used to ensure ornamental value year-round.

Design model 2 also allows the use of species such as *Trachelospermum jasminoides*, *Lonicera nitida* and *Passiflora caerulea*, thanks to the support structure. Cable systems are commonly used for sustaining fast growing plants with denser foliage, while wire-net systems are applied for slow growing plant species that require small grid intervals to ensure extensive coverage [46]. Using deciduous plants, such as *Vitis vinifera*, may cause the depreciation of the VGS due to leaf loss in autumn and winter. The main disadvantage of this VGS model is the potential lack of aesthetic appeal caused by the uneven and slow growth of plants. Direct and indirect systems of green façades provide almost the same benefits relating to building heating, energy saving for cooling and temperature decrease [40], but they are considered the least effective VGS to achieve benefit for noise reduction due to the lack of the growing media in close proximity to the building façade that is mainly responsible of sound insulation [37]. In most cases, vertical irrigation and drainage systems are not required, because plants are placed at the basement of building façade. Manual irrigation is sufficient to maintain the VGS, however drip, sprinkler or wicking irrigation can be installed in the planter box if automatic watering is required.

### 3.3. Design Model 3: Modular Panel System

**Figure 5:**
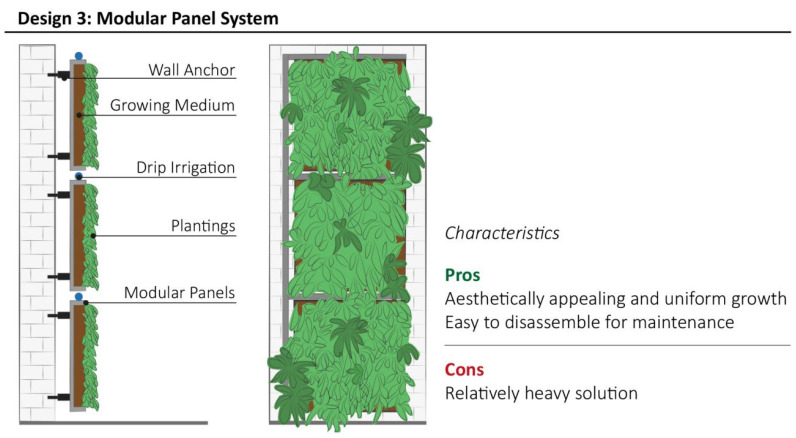
Schematic structure of Modular Panel System with the most relevant characteristics highlighted by stakeholders during interviews.

Design model 3 represents a modular pre-vegetated panel, based on commercial style products. Commonly, this type of VGS is characterised by a structural waterproof box panel (e.g., polystyrene or HDPE) that often contains an inorganic (e.g., mineral wool, felt or perlite) or organic (soil, potting mix) light-weight substrate, wrapped in a geotextile and equipped with a fertigation system (Figure 5) [42]. Alternatively, DIY designs of this type can be obtained by upcycling old wood pallets as demonstrated by) Pruitt et al. [47]. For those systems that are hydroponic, regular and automatic watering and fertilization is required [48], especially for inorganic substrate panels, to sustain vegetation growth. Additionally, drainage systems must be designed at the basement of the VGS. 

The waterproof insulation of VGS panels is mandatory to preserve the integrity of building façades from moisture [49]. Modular panel systems are designed to be anchored to the building through a support frame creating a void space between the panel and the surface, providing a better thermal performance than other VGSs [50] and enhanced noise insulation [51]. Due to the versatile structure, Design model 3 is suitable for rapid coverage of whole or part of large building surfaces [35]. This design system supports a wider group of evergreen plants, such as *Cholorophytum comosum*, *Sedum* spp., *Spathiphyllum wallisii*, *Epipremnum aureum* and other perennial or annual species for indoor and outdoor applications. Pre-vegetated systems ensure a high aesthetic result after installation, but maintenance is the key factor to preserve high ornamental appeal. 

### 3.4. Design Model 4: Textile Bag System

**Figure 6:**
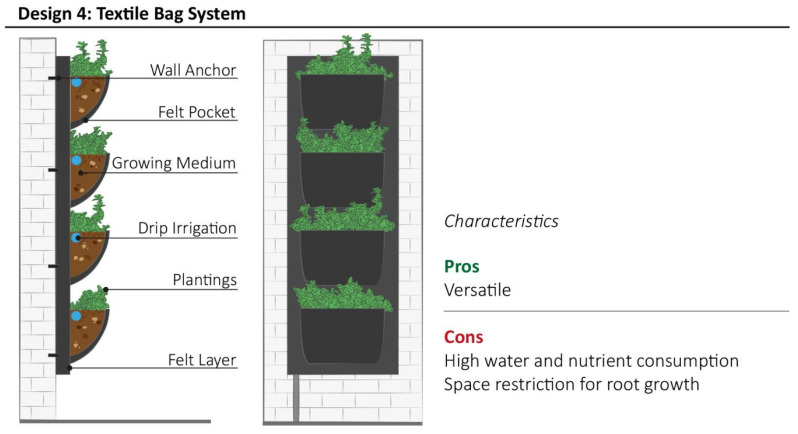
Schematic structure of Textile Bag System with the summary of most relevant characteristics highlighted by community representatives during interviews.

Design model 4 is constructed from a textile material, such as felt, geotextile, burlap, tarpaulin, or any other cloth strong enough to withstand water and weathering as well as the weight of the system itself (Figure 6). Plants and growing medium, such as soil, coconut fiber substrates, felt, expanded clay pellets, sphagnum or mineral wool, are usually contained within textile pockets. The fertigation system is selected based on the growing medium: wicking or subirrigation systems are more appropriate for felt or inorganic substrates, while surface drip irrigation or manual watering is suitable for soil-based systems. Excess irrigation water is drained from the system by cutting holes near the base of each pocket such as to provide optimal conditions for the plant species used. The main advantage of this VGS model is the lightweight nature of the structure, due to the extensive use of textiles, and the flexibility of application on sloped building surfaces. This VGS model may be used for large-scale projects or small-scale applications, such as domestic aromatic gardens, due to its modular structure. The main disadvantage of this VGS model is the lack of space for plant roots provided by the pockets. These pockets can contain plants such as small vegetables and aromatic herbs, rooted directly to the growing media or using the “root-wrapping system”, whereby roots are wrapped into a felt textile lightening the structure’s weight. Moreover, the modular pocket framework simplifies the replacement of plants during maintenance interventions.

### 3.5. Design Model 5: Planter/Pot System

**Figure 7:**
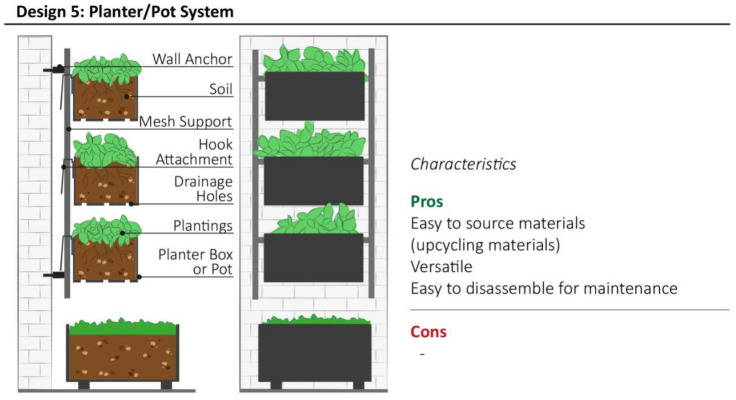
Schematic structure of Planter/Pot System with the summary of most relevant characteristics highlighted by community representatives during interviews.3.6. Design model 6: Guttering System.

Design model 5 consists of a modular VGS which utilises planter boxes or pots attached to a support structure. Components of this design model vary in shape, material, and structure. This VGS system is characterised by its use of relatively simple and common materials and components, adopted for multiple creative applications in indoor and outdoor environments (Figure 7). This design model is versatile and DIY systems are commonly created by upcycling materials such as plastic drink bottles [48] Depending on the structure and form of growing container, a large variety of shrub plants, aromatic herbs and edible plants can be cultivated in this model of VGS, while simultaneously providing high aesthetic value. Soil substrates are commonly used, but also light-weight substrates, such as coconut fibers, expanded clay pellets or sphagnum, can be added to reduce the whole system’s weight and to increase water drainage. Hand watering is recommended for small scale VGS of this design, while automatic or semi-automatic piped irrigation network is required for medium-large scale planter box systems. Depending on the structure of the VGS, a drip line irrigation network placed along the top of row is suitable for systems with planter boxes placed close to one other. Excess water can drain from each container by cutting holes at the base of planter boxes, while additional planter boxes can be placed at the base of the VGS to collect excessive water flow. 

**Figure 8:**
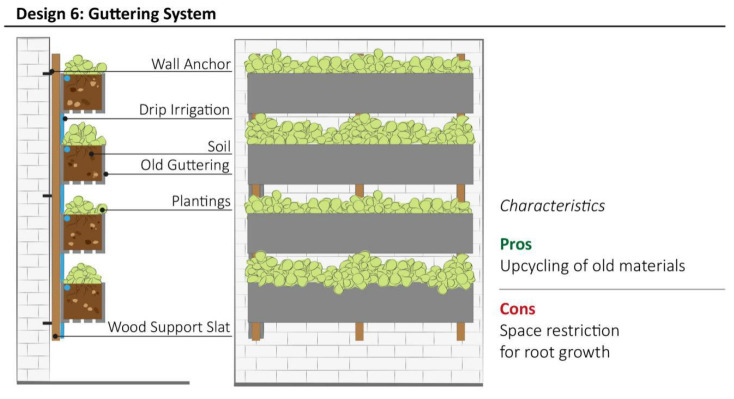
Schematic structure of Guttering System with the summary of most relevant characteristics highlighted by community representatives during interviews.

Design model 6 uses materials such as discarded rain gutters as vessels for growing plants, following the design proposed by Houz [52] This system is a creative DIY solution, and potentially the most cost effective VGS, which aims to improve sustainability by upcycling durable materials. A drip irrigation network can be installed along the top surface of the substrate, while excess water can easily flow downwards using gravity if gutters are placed on a slight angle. Otherwise, holes at the bottom of gutters can be cut for water drainage (Figure 8). The main disadvantage of this system is the limited depth and volume of growth substrate, thus limiting plant selection to those with shallow roots, such as succulents, strawberries and some ornamental plants. The aesthetic appeal of this system mainly depends by the creativity and ability of its makers in restoring old materials.

### 3.6. Design Model 7: Piping System

**Figure 9:**
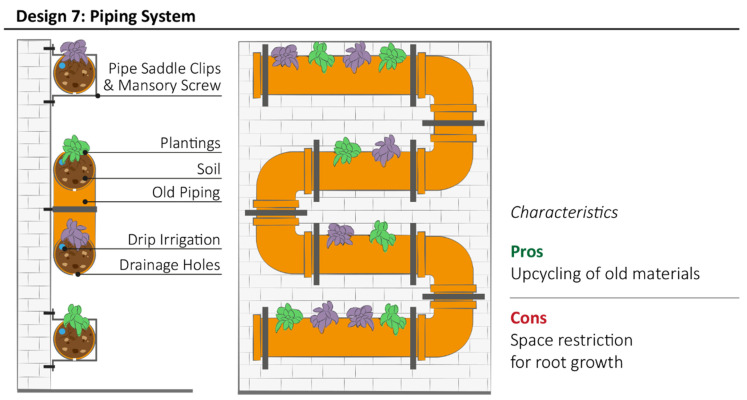
Schematic structure of Piping System with the summary of most relevant characteristics highlighted by community representatives during interviews.

Design model 7 uses old PVC pipes as the key structural component of the system, which can be directly attached to the wall through masonry screws and pipe saddle clips (Figure 9). Different pipe sections can be connected to each other through piping and plumbing fitting to obtain creative structures adapted to user needs. Pipes filled with a cultivation substrate (soil or other light-weight substrates) are used as vessels to hold the plant roots. Alternatively, planter pots—large enough for houseplants—may be placed into the piping cut outs, thus confining the cultivation substrate. Additionally, this method allows for easy cleaning and maintenance, as pots can be pulled out and replaced when needed. Depending on the size of the pots and pipes, this solution can be useful to increase the depth of the growing medium as pots can slightly extend past the piping edge. Edible plants, such as small vegetables, are commonly cultivated in piping systems. The linear structure allows an integrated drip irrigation system to be installed along each level. It is also recommended to drill drainage holes along the base of the horizontal pipes for excess water to seep through.

### 3.7. Design Model 8: Freestanding Wood-Based System

**Figure 10:**
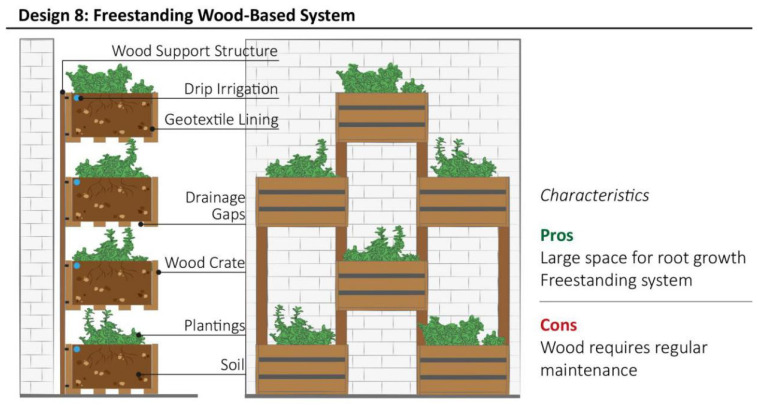
Schematic structure of Freestanding Wood-based System with the summary of most relevant characteristics highlighted by community representatives during interviews.

This system is structured using stacked wooden crates to enable vertical greening (Figure 10). Old wooden crates can be upcycled to hold growing media and plantings [53]. They are usually stacked and fixed together, using longer wooden planks as a support structure, allowing the unit to be freestanding. The structural dimensioning and a rough calculation of the system’s weight are essential to prevent overloading and collapse. The application of an external wood treatment, such as an oil-based finish, is necessary to avoid aesthetic and structural damage. Appropriate drainage systems and the use of porous and lightweight growing medium increase the wood’s durability and longevity. Moreover, the use of geotextile fabric to contain the growth medium and allow air and water to flow to through it may reduce the risk of moisture accumulation. Drip irrigation systems can be integrated into the vertical structure for regular watering. This model of VGS broadens the selection of plant species thanks to the growing medium volume provided by the wood crates.

### 3.8. Comparison between VGS Design Models

The summary of eight VGS design models’ characteristics is presented in Table 3. Comparing the above eight designs demonstrates the relative suitability and achievability each design has for a given application. Indications were obtained by integrating interview results and the analysis of scientific and grey literature.

**Figure 11:**
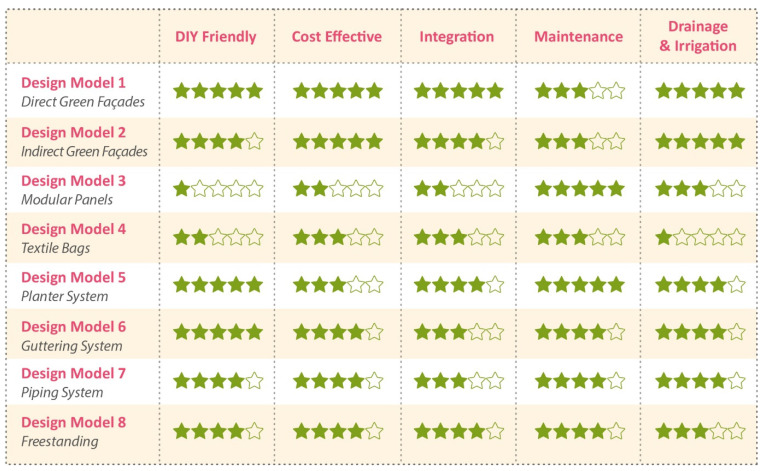
Star-based rating system that compares the VGS designs using community or domestic scale relevant criteria.

The star rating system is presented in Figure 11, comparing five essential design parameters of the eight VGS models, based on the engagement with the stakeholders of community garden groups interviewed and informed by their direct experience in designing, constructing and maintaining systems. Qualitative ranking was adopted to account for the stakeholders’ assertions and to provide an insight into local and specific community gardeners’ attitudes and challenges. Final scores concerning cost effective criteria shown in Figure 11 are not the results of the analysis of quantitative data collection because each community garden presents unique characteristics (such as dimensions and site) and strategies (e.g., using new or recycled materials) for implementing VGSs. Scores for each criterion are defined based on the specific experience of the seven case studies involved in the current research and they may change as the community members and locations differ due to the bottom-up attitude of community gardens and of Do-It-Yourself practice. The star rating system aims to provide an initial and non-site-specific assessment tool to guide communities into their first stages of the decision making process. Green façade designs obtained the highest scores, while more complex systems such as Modular Panels and Textile bag designs scored comparatively lower. 

## 4. Discussion

### 4.1. A VGS Design Model for Any Requirement

Whilst we have categorised VGSs into eight design models, each design can be modified or customised according to the specific requirements and motivation of each VGS project.

With the exception of Design models 3 and 4, the interview feedback deemed to require specific skills, technical knowledge, tools and materials, the other VGSs were deemed to be generally highly DIY friendly. According to the community group interviewees, Design model 1 and 2 are the simplest VGSs to implement, using relatively lightweight materials, which are easy to source. However, the aesthetic value of these systems is dependent on the vegetation used [54], as some species may take longer to grow and spread than others. Due to the limited number of system components, direct and indirect green façades are easily integrated with building surfaces [55]. According to the community group interviewees, Design model 5 includes a wide range of creative solutions that can be easily integrated in different contexts due to its structural modularity and versatility. Design models 6, 7 and 8 are highly DIY friendly, but the freestanding model requires specific technical skills during assembly to ensure structural stability, while the other VGSs are anchored to the building’s indoor or outdoor surface. Despite this, a key advantage of freestanding VGS is the boxed system allowing for a larger volume of soil to be used compared to the planter boxes, piping and guttering systems [56]. Another important positive feature of Design model 8 concerns the easy integration into indoor and outdoor spaces, because it does not require a supporting building wall, providing it with an alternative use as a vegetated screen.

Due to their simplicity in design and lack of structural requirements, green façades (Design models 1 and 2) were determined by community garden representatives to be the most cost-effective solutions. These systems were also relatively easy to maintain compared to living walls. On the other hand, Design model 3, which has greater perceived ornamental value by the community garden representatives, is the most expensive VGS solution due to its structural complexity. These VGSs are commonly supplied by companies expert in vertical greening and urban landscaping which also provide supervision for the installation [57]. The cost of this type of vegetated system will differ depending on factors including the supplier and manufacturer, installation requirements and the size of the system. Thus, Design model 3 is less budget or DIY friendly than the other systems tested here, and for this reason modular vegetated panels are usually adopted by end users able to support their higher initial costs. Design model 5 is a relatively cost-effective solution especially if planter pots are obtained by upcycling waste materials, such as plastic bottles and containers. 

Considering that no VGS is completely maintenance free, Design models 3 and 5 are the easiest systems to maintain according to interview respondents, due to their modular structure that facilitates the replacement of components when required. Nonetheless, structural maintenance on commercial modular panel systems is commonly carried out by specialized experts (Design model 3). Direct and indirect green façades (Design models 1 and 2) require only basic maintenance, such as ongoing pruning and general plant care due to the type of climbing vegetation used. Other VGS design models present similar maintenance requirements, while the use of textile materials for Design model 4 may hinder the replacement of damaged parts and complicate routine maintenance to a degree. 

The automated irrigation and drainage systems are the most critical components for VGSs, excluding direct and indirect green façades that are most commonly manually watered. Community group stakeholders’ comments indicated that Design model 4 requires a more complex drainage and irrigation system than other VGSs due to the textile material and pocket structure. Moreover, Design models 3 and 4 are commonly set up as hydroponic systems that require a fertigation system for supplying nutrients to their inorganic plant growth substrates. Some form of drainage system is an essential component for all types of VGS to preserve the system’s structure and vegetation health. Regardless of the type of irrigation and drainage systems used, all wooden surfaces and structural components should be treated with waterproof finish to increase product life. Moreover, all VGSs that are located in indoor environments require a reliable drainage system with a tank for excess water collection [58]. This aspect is particularly important for freestanding VGSs as they are commonly placed in indoor spaces where water leakage may present a serious safety hazard or risk to property damage. 

### 4.2. Implementing DIY Vertical Greening with Communities in Real Setting

In order to support stakeholder co-design or participatory design practice, some preliminary evaluations should be considered regarding the motivation for wanting a vertical greening system, features of the selected site and the skills, time commitment and abilities of the community before choosing the most appropriate VGS [59]. The pivotal aspect for the successful implementation of a VGS will be accurate identification of available resources, skills and goals, which will be different for each installation also for each location and the different kind of people involved linked to their motivation.A co-design strategy should be applied with the purpose of: (1) sharing the motivation framework and knowledge concerning VGS amongst the stakeholder community members; (2) identifying barriers that could create a gap between the ideal project goal and practical implementation; (3) identifying resources and strategies to address this gap. Focus groups and dedicated workshops could be organized in order to facilitate the identification of the main drivers for co-designing the most affordable VGS according to community motivation and goals [60]. The definition of community motivation is the first important step to establish which type of VGS is the most appropriate for a given community’s purpose. It is important for communities to identify what their needs, requirements and limitations are before starting to design a VGS. All communities are different; therefore the choice of design should capture the wide range of different levels of DIY ability, budget, time availability and resources. VGSs models can be customized based on the technical abilities of community members and the available budget to implement the project. The evaluation of community resources is particularly important for selecting appropriate cost-effective solutions for vertical greening, such as choosing to buy new products that include the provision of expert advice or the use of recycled materials. Moreover, it is also necessary to consider the time and commitment that the community’s members can allocate to daily maintenance.

It is the authors’ perspective that site selection should take into account the community’s needs, limitations and how VGS specific design aspects will interact with the site before deciding the most appropriate VGS. The site location and orientation to sunlight, climate conditions for outdoor VGSs, the suitability of an existing wall structure for green wall retrofit, water provision, local regulations and the need for professional advice should be analysed before designing a VGS [61]. These design drivers can guide and facilitate the design of successful vertical greening solution. Elements of different systems can be combined to optimize the design and satisfy the community’s needs, whilst complying with the site’s limitations. There is not a ‘one size fits all’ approach for developing, designing and maintaining all types of vertical greening solutions [27].

### 4.3. Comparison between Commercial VGS and DIY Design Models

This work has showcased eight design models of VGSs capable of being installed by those without expertise in the field, however the degree of complexity in design and maintenance requirements is dependent on the design type. Some VGSs do not require any specific abilities, such as Design models 1 and 2, while others are reliant on specific materials and technical skills to construct them. Several commercial companies offer ready-to-use solutions and materials for simple design models which can facilitate DIY VGS installations. For example, multistory commercial systems may now offer wire trellis systems specifically designed to implement indirect green façades to cover wide building surfaces [62]. Nevertheless, support structures for small-scale or domestic VGSs can be easily constructed by recycling or upcycling disposed materials, such as sticks, nets and ropes.

The feedback from stakeholders identified Design model 3 as the most complex and challenging VGS to construct using DIY practice. It is thus unsurprising that several companies sell system components for this Design model type, which can help to bridge the gap between expert and inexpert VGS installers. Numerous commercial products of this design are available, with providers frequently offering assistance with the system installation [63], while other companies, offer DIY vertical garden kits provided with inorganic cultivation substrates and planter panel or boxes with holes in which to place plants for indoor applications. Construction challenges also apply to Design model 4, because it requires specific sewing skills. 

Design model 5 is the most versatile and creative VGS, as a wide range of products can be used as planter boxes. Nonetheless, some companies, offer commercial products based on modular planter box structure, and support clients during the installation process. Available commercial products are likely to be more durable and stable, and they are recommended in contexts that require high surface coverage. However, more creative solutions are suggested to increase public engagement in the design and installation processes and to improve sustainability through upcycling materials.

Community garden groups most commonly prefer Design models 6, 7 and 8 which are DIY friendly and low-cost to implement and maintain. They are composed of recycled and upcycled materials, reflecting community gardens values on sustainability. 

## 5. Conclusions and Future Directions

The findings from this study contribute to reducing the lack of DIY technical information related to VGS design choice and installation and indicate critical considerations that can arise during VGS implementation in small-scale urban spaces. The UN SD Goal 11 encourages the growing trend and common interest in urban vertical greening that should be supported by appropriate knowledge for beginners.

The involvement of stakeholders with expertise in community garden activities through informal interviews enabled the collection and organisation of information useful for DIY applications. In order to make VGSs as DIY urban interventions more accessible, stakeholder experience was used to define a user-friendly interpretation of vertical greening technology that has, in most cases, previously been described within a scientific and academic mindset. Knowledge dissemination about the importance of VGSs as green infrastructure and about their construction plays a pivotal role for community engagement in making more vertical greening and in promoting the participatory transition towards more sustainable and green cities.

Future work is required, and should focus on real world in situ examples, in order to provide concrete evidence and truly quantify the outcomes of this work in specific contexts. Given the great variability of building types and settlement systems where VGSs can be implemented, it is recommended that the applications of this technology, and the quantification of its success, be determined in as many specific building-districts as possible, so that the current evaluation model can provide more comprehensive information through revision [64].

Additionally, it is suggest that future work investigate if the methods used in this study be used to support cities with food through urban agriculture, and how much food could produce, as there is a here is a growing interest in making such systems dual purpose for food production. Such usage presents challenges related to the fate of air pollutants within urban environments, and whether this will affect the quality of food so produced. Significant further research will be required before these new systems can be used with confidence for food production. Similarly, research that enables the comparison of a food supply cultivation and the energy and water requirements can also be explored (Water-Energy and Food nexus).

## Figures and Tables

**Table 1:** Details of interviews to Community Garden Groups.

	Stakeholder	Location
1	Turramurra Comunity Garden	Turramurra, NSW
2	Coal Loader Community Garden	Waverton, NSW
3	Milson Community Garden	Milson Point, NSW
4	Glovers Garden	Lilyfield, NSW
5	Wentworthville Community Garden	Wentworthville, NSW
6	Taringa St Community Garden	Ashfield, NSW
7	Ultimo Community Garden	Ultimo, NSW

**Table 2:** Questions used to guide stakeholders of community gardens into scoring each criterion for VGS models evaluation according to their experience. Each criterion is described using significant questions that have the same weight. The final score of each criterion is obtained by the arithmetic mean of the values assigned to each question.

Criterion	Description	Rating Range
DIY friendly	How easy is the VGS to DIY? How easily can the materials be sourced? Is it possible to use recycled/repurposed materials and components? How easy is the assembly? Can the VGS be designed, developed and constructed without the supervision of an expert or professional?	One star (not DIY friendly) to five stars (very DIY friendly)
Cost effective	How budget friendly is the VGS? Are there operational costs during the VGS’s life cycle? Is it possible to reduce cost using recycled materials and components?	One star (least cost effective) to five stars (most cost effective)
Integration with existing buildings	How easy is the VGS to implement onto a vertical surface? Is the VGS adaptable to different sized and shaped surfaces? Does the VGS need any specific structural support?	One star (not easy to integrate) to five stars (easy to integrate)
Maintenance	How easy is the VGS to maintain? How frequent is the required maintenance? Must experts carry out maintenance? How many people are required for maintenance?	One star (not easy to operate and maintain) to five stars (easy to operate and maintain)
Drainage and Irrigation	How complex is the drainage and irrigation system to install and operate? How many components does the irrigation system require? Does the VGS need a fertigation system?	One star (complex drainage and irrigation) to five stars (simple drainage and irrigation)

**Table 3:** Schematic description of VGS design models based on selected six criteria.

	Structure, Materials & Components	Irrigation and Drainage Systems	Vegetation	Indoor/Outdoor Application	Maintenance	Aesthetic Value
Design 1	None	Manual	Self-clinging climber plants	Outdoor	Routine: pruning to stimulate or avoid excessive growth	Low: non-homogenous surface coverage
Design 2	Vertical support structure (trellis, mesh, nets, wires or cables) in bamboo, wood, steel, aluminium or HDPE Wall anchors Planter box	Manual or automated and integrated into the planter box	Climbing plants	Outdoor	Routine: pruning to stimulate or avoid excessive growth Check the status of vertical support	Low
Design 3	Panel in HDPE, polystyrene, or wood Waterproof screen (PVC) to preserve surface building Growing medium: inorganic light-weight substrate (e.g., mineral wool, felt or perlite) or organic (soil) Wall anchors Wall support structure (steel rod or trellis)	Hydroponic system: Ferti-irrigation system using drip, sprinkler, or wicking irrigation systems Automated and integrated into the panel Drainage system to collect excessive water	High variety of evergreen and ornamental plant species	Outdoor & Indoor	Watering and fertilization Replacing plants Clearing fallen debris Cleaning ferti-irrigation system Check the status of panels and waterproof screen	High: large and flexible vegetation surface coverage
Design 4	Textile bag system in felt, geotextile, old burlap, tarpaulin Waterproof screen (PVC) to preserve surface building Growing medium: inorganic light-weight substrate (e.g., felt substrate, expanded clay pellets, sphagnum or mineral wool) or organic (soil or coconut fibre) Geotextile to contain growing medium Wall anchors Wall support structure (steel rod or trellis)	Manual or surface drip irrigation for organic substrates Wicking or integrated ferti-irrigation system for inorganic substrates Drainage system: holes at the base of each pocket	Small vegetables Aromatic herbs Ornamental plants	Outdoor & indoor (indicated for domestic-scale aromatic gardens)	Watering and fertilization Replacing plants (facilitated by pocket-based system) Cleaning ferti-irrigation system Check the status of textile material and of waterproof screen	Medium: depends mainly by the quality of textile bag system
Design 5	Pots, planter boxes, plastic bottles Growing medium: soil mixed with coconut fibre, expanded clay pellets and sphagnum Hook attachments Wall anchors Wall mesh support structure (steel rod or trellis)	Manual watering for small-scale systems Automated ferti-irrigation for large-scale system (dripline irrigation network) Drainage system: holes at the base of each planter box	Wide range of plants cultivated (ornamental, herbs and edible) depending by the planter box’s size	Outdoor & indoor	Routine: pruning, watering and checking the status of irrigation system	High vegetation coverage Creative solutions
Design 6	Rain guttering Growing medium: soil Wall anchors Wall mesh support structure (steel rod or trellis)	Manual watering Surface automated drip irrigation network	Plants with shallow roots, such as succulents or some ornamental plants	Mainly outdoor application	Routine: pruning, watering and checking the status of irrigation system	Creative solution
Design 7	Old pipes Pots (optional) Growing medium: soil Wall anchors	Automated and integrated drip irrigation network (also sprinkler)	Edible plants: small vegetables and herbs Ornamental plants	Mainly outdoor application	Routine: pruning, watering and checking the status of irrigation system	Creative solution
Design 8	Wooden crates Vertical support structure of wooden planks (for self-sustaining system) Growing medium: soil mixed with coconut fibre, expanded clay pellets and sphagnum	Manual Automated and integrated drip irrigation network	Wide range of plant species cultivated	Outdoor & Indoor (indicated for dividing spaces and preserving social distance)	Routine: pruning, watering and checking the status of irrigation system Occasionally: checking the status of wood components	Creative and flexible solution
